# Exudative pharyngitis and *Corynebacterium pseudodiphtheriticum*: A case report and review of the literature

**DOI:** 10.4102/sajid.v36i1.225

**Published:** 2021-03-29

**Authors:** Kessendri Reddy, Sebastian Gericke, Helena Rabie, Colette Pienaar, Motlatji Maloba

**Affiliations:** 1 Department of Microbiology, National Health Laboratory Services Tygerberg, Cape Town, South Africa; 2 Division of Medical Microbiology and Immunology, Department of Pathology, Faculty of Medicine and Health Sciences, Stellenbosch University, Cape Town, South Africa; 3 Department of Paediatrics and Child Health, Faculty of Medicine and Health Sciences, Stellenbosch University, Cape Town, South Africa; 4 Division of Paediatric Infectious Diseases, Department of Paediatrics and Child Health, Faculty of Medicine and Health Sciences, Stellenbosch University, Cape Town, South Africa; 5 Department of Medical Microbiology, Faculty of Health Sciences, University of the Free State, Bloemfontein, South Africa

**Keywords:** exudative pharyngitis, *Corynebacterium pseudodiphtheriticum* clinical presentation, *Corynebacterium pseudodiphtheriticum* laboratory identification, *Corynebacterium pseudodiphtheriticum* disease spectrum, emerging pathogen

## Abstract

*Corynebacterium pseudodiphtheriticum* is an established member of the normal flora of the respiratory tract. This organism is an emerging cause of respiratory tract infection, as well as infection of the skin and skin structures, urinary tract and other sterile sites. The syndrome of *C. pseudodiphtheriticum* exudative pharyngitis is a diagnostic challenge of particular relevance in recent times as this organism can be confused with *Corynebacterium diphtheriae* in the clinical setting and in the laboratory. We report a case of exudative pharyngitis, possibly due to *C. pseudodiphtheriticum*, in a 14-month old, incompletely vaccinated, human immunodeficiency virus (HIV)-positive infant and review the role of this organism in terms of its microbiological profile and identification, disease spectrum and antimicrobial susceptibility pattern.

## Background

*Corynebacterium pseudodiphtheriticum* has been described as an emerging respiratory pathogen and forms part of normal upper respiratory tract flora.^1^ This organism has been linked to pulmonary disease in patients with underlying lung abnormalities, chronic medical conditions and immunocompromised states, including human immunodeficiency virus (HIV),^1,2,3^ although disease is also described in immunocompetent patients. Respiratory tract disease caused by this organism is likely to be underestimated, given its role as a respiratory tract commensal.

We report a case of exudative pharyngitis potentially caused by *C. pseudodiphtheriticum* in an unvaccinated HIV-positive infant. There have been a handful of cases describing exudative pharyngitis in conjunction with *C. pseudodipththeriticum* to date; this case provides further evidence of its possible aetiological role and highlights the need for its inclusion in the differential diagnosis of diphtheria. This case is of particular relevance in the current climate of reduced vaccination uptake globally, when clinical acumen surrounding the syndrome of exudative pharyngitis should be maintained.

## Clinical case

### Presentation

A 14-month-old, previously well boy presented to a district hospital in the Western Cape in December 2016 with respiratory distress related to upper airway obstruction. The boy was moderately acutely malnourished and newly diagnosed with HIV on admission.

The child presented with a short history of fever, dysphagia and lethargy. He had a household contact with pulmonary tuberculosis (TB); no TB chemoprophylaxis was given. He had no travel history. Vaccination status was incomplete, with only birth vaccinations administered (Bacille Calmette Guérin [BCG] and oral polio vaccine).

**FIGURE 1:**
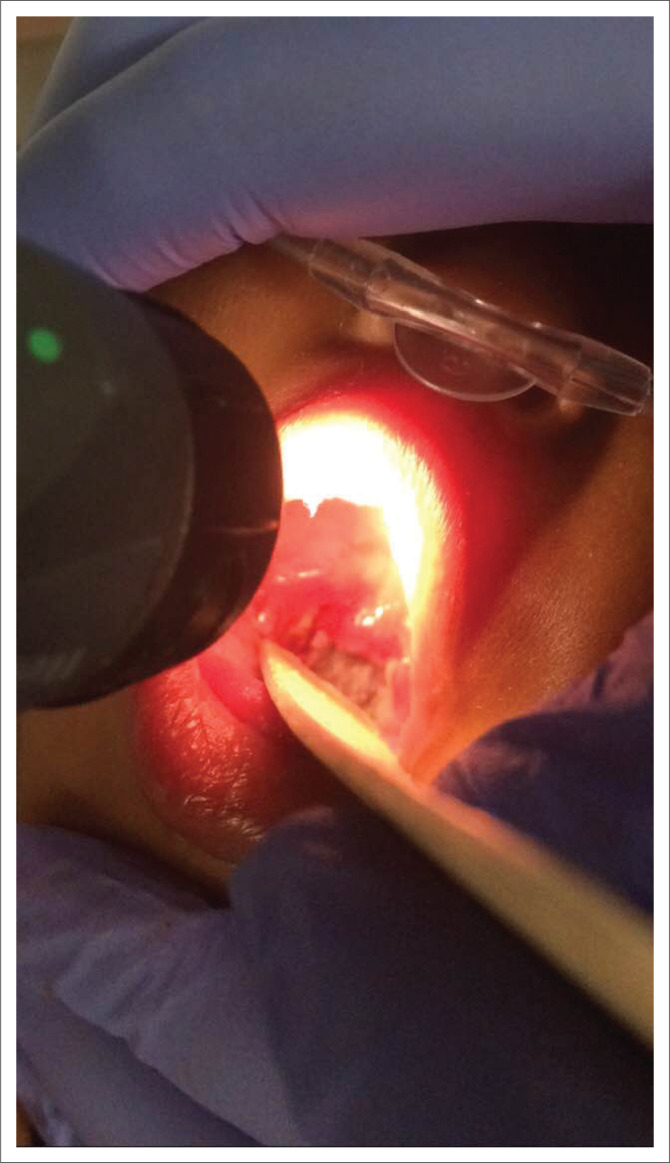
White membrane adherent to the pharynx noted on oropharyngeal examination on presentation in a 14-month-old incompletely vaccinated infant with respiratory distress.

On examination, the child was in shock and had respiratory distress with drooling, forward posturing, grunting and pulsus paradoxus. Oropharyngeal examination revealed an inflamed and oedematous uvula and tonsils with a thick, white, friable membrane adherent to the oropharynx which bled upon touch (Figure 1), prompting a provisional diagnosis of diphtheria.

No neck swelling, cervical lymphadenopathy, cranial nerve palsies or muscle weakness was present. Examination of the chest was not suggestive of lower respiratory tract infection.

Potential aetiological considerations included the following: group A streptococcus (*Streptococcus pyogenes)*, group C or G streptococcus, diphtheria, *Arcanobacterium haemolyticum*, adenovirus, Epstein–Barr virus (EBV), acute HIV infection and candidiasis.

### Management

Fluid resuscitation, supplemental oxygen and adrenaline nebulisations were provided.

Ceftriaxone (a third-generation cephalosporin) was given intravenously along with paracetamol. The child was intubated because of worsening respiratory distress and transferred to Tygerberg Hospital’s Paediatric Intensive Care Unit. Diphtheria antitoxin could not be sourced, and was omitted from patient management.

Infection control precautions were followed as for diphtheria whilst awaiting confirmatory results. Standard precautions were combined with droplet precautions, including patient isolation, glove and apron use, and the use of facemasks for both the patient and healthcare providers.

### Investigations

His white cell count was 1.4 × 10^9^/L, haemoglobin 9.0 g/dL and platelet count 54 × 10^9^/L. The serum C-reactive protein (CRP) was 258 mg/L. An HIV-1 antibody test and polymerase chain reaction (PCR) performed on admission were positive. His absolute CD_4_ (cluster of differentiation 4) count was 356 cells/µL (29%). An HIV viral load was not performed.

Epstein–Barr virus serology supported previous exposure (EBV nuclear antigen IgG positive, EBV viral capsid antigen IgM negative). Herpes Simplex Virus 1/2 and adenovirus PCR were negative.

Blood cultures revealed no growth.

A tracheal aspirate on admission showed occasional non-branching small Gram-positive bacilli in Chinese letter formation and no neutrophils on Gram stain, and revealed no growth after 48 h in a 5% CO_2_-enriched incubator. A nasal swab taken on admission showed a pure growth of *Corynebacterium* colonies. This sample was auramine O smear-negative for TB and negative on Xpert MTB/Rif testing (Cepheid, Sunnyvale, California).

Two throat swabs taken in the first 24 h of admission (one prior to antibiotics) showed no pathogens after 72 h of incubation. The throat swabs and a nasal swab were also cultured and tested molecularly at the National Institute for Communicable Diseases and were culture-negative and PCR-negative for *Corynebacterium diphtheriae* toxin. No membrane tissue samples were collected.

### Outcome and follow-up

The patient improved steadily on a 14-day course of ceftriaxone. Antiretroviral therapy and catch-up vaccinations^4^ were commenced on discharge.

### Public health response

Nasal and throat swabs were collected from 36 asymptomatic household and healthcare contacts. *Corynebacterium pseudodiphtheriticum* was isolated from the household contacts only: the index patient’s mother and two children in the household (aged 4 and 9 years). These contacts received a 5-day course of azithromycin.

## Laboratory identification of *Corynebacterium pseudodiphtheriticum*

Throat and nasal swabs were inoculated onto tellurite-containing and routine media and incubated at 35 °C in ambient and 5% CO_2_-containing incubators, respectively; colony growth was better in the CO_2_-enriched atmosphere. Grey-black colonies suspicious of *C. diphtheriae* were observed on tellurite-containing media after 48 h incubation, with corresponding pure growth of white, butyrous colonies on tryptose blood agar on the nasal sample only. Gram stain of these colonies revealed non-branching, uniform Gram-positive bacilli.

Neither beta-haemolytic colonies nor yeasts were cultured from any of the samples.

Biochemical identification with the VITEK(R) 2 ANC system (bioMérieux, Marcy l’Etoile, France) confirmed by the BD BBL Crystal™ Gram Positive ID Kit (Becton Dickinson and Company, Franklin Lakes, USA), convincingly identified *C. pseudodiphtheriticum*. Diphtheria toxin was not detected molecularly or phenotypically (in-house PCR negative at the National Institute for Communicable Diseases, Elek test negative).

Limited susceptibility testing was performed using the gradient-diffusion based E-test method (bioMérieux, Marcy l’Etoile, France). Results were interpreted using *Corynebacterium* species criteria (Clinical and Laboratory Standards Institute M45 document, 2015) and are summarised in Table 1.

**TABLE 1:** Antimicrobial susceptibility testing results of the *Corynebacterium pseudodiphtheriticum* strain isolated on nasal swab from a 14-month-old infant presenting with exudative pharyngitis.

Antimicrobial agent tested	Minimum inhibitory concentration (μg/mL)†	Interpretation category for *Corynebacterium* species, CLSI
Penicillin	0.012	Susceptible
Cefotaxime/Ceftriaxone	0.047	Susceptible
Ciprofloxacin	0.094	Susceptible
Vancomycin	0.19	Susceptible
Azithromycin	0.125	-

CLSI, Clinical and Laboratory Standards Institute M45 document, 3rd edition 2015.

† , Minimum inhibitory concentration obtained by gradient diffusion testing (Etest, bioMérieux, Marcy l’Etoile, France).

## Ethical consideration

Informed consent was obtained from the child’s mother and ethical approval was granted by Stellenbosch University’s Human Health Research Ethics Committee (Reference number C20/01/002).

## Discussion

*Corynebacterium pseudodiphtheriticum* is an aerobic non-spore-forming Gram-positive bacillus, forming part of the upper respiratory tract flora.^1,2^ Recent studies have investigated the probiotic role of *C. pseudodiphtheriticum* in reducing nasal colonisation with *Staphylococcus aureus*,^5,6^ *Streptococcus pneumoniae* and respiratory syncytial virus.^7^

*Corynebacterium pseudodiphtheriticum* has also been associated with respiratory tract infection, including pneumonia, bronchitis and single cases of necrotising tracheitis and a lung abscess.^3,8,9^

These infections have largely been noted in patients who are immunosuppressed, such as HIV-positive patients or transplant recipients; patients with underlying lung disease, such as chronic obstructive airways disease; and patients with underlying medical conditions, such as congestive cardiac failure, ischaemic heart disease and malignancy.^1,2,3,10^ Patients with cystic fibrosis may be particularly at risk.^11^

Pulmonary infection has also been linked to invasive respiratory procedures such as endotracheal intubation, presumably because of direct introduction of this commensal into the lung.^12^ Recent data suggest that previous exposure to antimicrobials favours colonisation with this organism.^1^ The few cases of lower respiratory tract infection reported in immunocompetent patients suggest that an older age may play a role^3,8^ but this has been challenged.^13^

Co-infection with recognised respiratory tract pathogens has been reported^2,3,11,12,13^ and a co-pathogenic role theorised.^10^ Although carriage of these organisms can result in sample contamination, microbiological methods to determine significance include moderate-to-substantial palisading Gram-positive bacilli^12^ or diphtheroids within polymorphonuclear leucocytes on Gram stain,^3^ and substantial growth of diphtheroids on culture,^10^ in conjunction with clinical presentation and underlying risk factors.^2^

Other infectious processes associated with *C. pseudodiphtheriticum* include infective endocarditis,^14,15^ keratitis,^1^ endophthalmitis,^1^ skin infection,^16^ urinary tract infection,^13^ osteitis and septic arthritis.^13,17,18^ To our knowledge, three cases of exudative pharyngitis because of *C. pseudodiphtheriticum* have been reported to date, as summarised in Table 2.

**TABLE 2:** A summary of important clinical features in reported cases of *Corynebacterium pseudodiphtheriticum* exudative pharyngitis to date (June 2020).

Variable	Case 1 – 1996†	Case 2 – 1997‡	Case 3 – 2014§
Age	32-year old	4-year old	6-year old
Gender	Male	Female	Female
Presenting symptom	Sore throat, dysphagia, fever	Fever, generalised lymphadenopathy	Fever, sore throat, neck swelling, nasal obstruction, toxic
Oropharynx	Greyish-white exudate from tonsils to posterior pharyngeal wall, enlarged tonsils, tender cervical lymphadenopathy, erythema and oedema	Greyish-white membrane attached to posterior pharyngeal wall, erythema	White, leathery membrane over tonsils, congestion, cervical lymphadenopathy
Underlying history	Not reported	Not reported	Not reported
Treatment	Penicillin IM, diphtheria antitoxin	Cefprozil and erythromycin	Penicillin IV, diphtheria antitoxin
Outcome	Cured	Cured	Cured
Immunisation history	‘All’ received in childhood	Not immunised	Fully immunised

Note: Please see the full reference list of the article, Reddy K, Gericke S, Rabie H, Pienaar C, Maloba M. Exudative pharyngitis and *Corynebacterium pseudodiphtheriticum*: A case report and review of the literature. S Afr J Infect Dis. 2021;36(1), a225. https://doi.org/10.4102/sajid.v36i1.225, for more information.

IM, intramuscular; IV, intravenous.

† , Santos et al.^19^;

‡ , Izurieta et al.^20^;

§ , Indumathi et al.^21^

Despite the fact that *C. pseudodiphtheriticum* has traditionally been described as non-toxigenic,^22^ we believe confirmation of this is advisable in view of the mechanism of transmission of diphtheria toxin. Diphtheria toxin is carried on a β-phage that infects bacterial cells belonging to the species, *C. diphtheriae, C. ulcerans* and *C. pseudotuberculosis.* Clinical experience suggests that this toxin does not affect other *Corynebacterium* species, although this is not impossible.

The identification of *Corynebacterium* species can prove complex, adding to the laboratory’s diagnostic dilemma.^23^ The gold standard for laboratory identification is sequencing of the hypervariable *rpoB* gene, which is not routinely available. Sequencing of the 16S ribosomal RNA gene is more widely available but may be inferior to partial *rpoB* sequencing,^24^ although database updates may have addressed this.

Common, presently available biochemical/proteomic methods to identify *C. pseudodiphtheriticum* in South Africa include:

- VITEK^®^ 2 ANC (bioMérieux, Marcy l’Etoile, France): *C. pseudodiphtheriticum* correctly identified in nine isolates identified on 16S rRNA sequencing.^25^
- BD BBL Crystal™ Gram Positive ID (Becton Dickinson and Company, Franklin Lakes, USA): Correctly identified 7 of 12 control *Corynebacterium* isolates to species level, misidentified one *Corynebacterium* species as *C. pseudodiptheriticum.*^26^
- API^®^ Coryne version 4.0 (bioMérieux, Marcy l’Etoile, France): Correctly identified one control strain as *C. pseudodiphtheriticum*^26^ and overall correct identification in 97.7% of the 1880 *Corynebacterium* species tested.^27^
- Vitek MS (bioMérieux, Marcy l’Etoile, France): 77% of 114 *Corynebacterium* isolates with species-level identification correctly identified, including one *C. pseudodiphtheriticum* strain.^28^

*Corynebacterium pseudodiphtheriticum* is usually susceptible to penicillin, vancomycin, rifampicin and the aminoglycosides,^2,29^ but geographical variability may be marked. A recently published study from Canada reported *C. pseudodiphtheriticum/C. propinquum* as displaying the lowest minimum inhibitory concentration (MIC) to penicillin.^29^ In contrast, penicillin resistance was reported in 38.9% – 44.2% of *C. pseudodiphtheriticum* strains from a variety of anatomical sites in Brazil in 2009; 15.0% of these strains were multidrug-resistant (resistant to more than 10 antimicrobial agents).^13^ In general, *Corynebacterium* species other than *C. diphtheriae* have become less susceptible to the beta-lactams in the last two decades.^29^ In this patient, de-escalation from ceftriaxone to penicillin was not possible because of a shortage of penicillin.

Macrolide and lincosamide resistance occurs consistently,^1,2,10,12,29,30^ which is concerning as erythromycin and azithromycin are recognised alternative options in the management of the clinical syndrome of diphtheria.^31^ Quinolone and trimethoprim-sulphamethoxazole resistance is variable.^3,8,13,29^ Susceptibility rates to tetracycine, linezolid and daptomycin are high.^29^ In this case, the low azithromycin MIC was reassuring for the prophylactic use of this agent in the carriers identified, although the clinical significance of carriage in this context is uncertain.

The limitations of this case study include the isolation of this organism from only a single admission nasal swab with a suggestive but unproven causative link and the absence of comprehensive antibiotic susceptibility testing. We did not assess strain relatedness between isolates, although the absence of this organism from any other contacts screened increases the likelihood of these being genetically related. The production of a novel toxin producing similar effects to diphtheria toxin was not explored and could be researched in future studies.

## Conclusion

This case adds evidence to the likely role of *C. pseudodiphtheriticum* as an emerging pathogen and highlights diagnostic dilemmas faced both clinically and microbiologically. The direct causal link was not present, but the absence of an alternative explanation for the clinical syndrome lends more weight to the possible aetiological role of this organism in exudative pharyngitis. Local susceptibility data are needed, as are further reports exploring the significance of isolation of this organism in a South African setting with our high prevalence of HIV infection. It is important that this organism is considered in the workup of exudative pharyngitis although, until its role is clarified, emphasis should remain on excluding diphtheria in the current climate of suboptimal vaccination coverage and anti-vaccination sentiment.
